# HASCom: A Heterogeneous Affective-Semantic Communication Framework for Speech Transmission

**DOI:** 10.3390/s26072158

**Published:** 2026-03-31

**Authors:** Zhenjia Yu, Taojie Zhu, Md Arman Hossain, Zineb Zbarna, Lei Wang

**Affiliations:** 1 Portland Institute, Nanjing University of Posts and Telecommunications, Nanjing 220003, China; 2 School of Communication and Information Engineering, Nanjing University of Posts and Telecommunications, Nanjing 220003, China; 3 College of Overseas Education, Nanjing University of Posts and Telecommunications, Nanjing 220003, China

**Keywords:** lowercase communication, emotional speech, heterogeneous transmission, diffusion model

## Abstract

Driven by the development of next-generation wireless networks and the widespread adoption of sensing, communication is shifting from traditional bit-level transmission to intelligent, rich interactions within our digital social system. However, existing speech semantic communication frameworks predominantly focus on textual accuracy, neglecting the critical affective information (e.g., tone and emotion) that is essential for natural human-centric interactions in the real world. To address this limitation, we propose the Heterogeneous Affective Speech Semantic Communication (HASCom) framework, designed for the robust transmission of highly expressive speech over complex wireless channels. Specifically, we design a heterogeneous dual-stream transmission architecture that decouples discrete phoneme-level linguistic content from continuous emotional embeddings. For discrete semantic information, we use reliable digital coding protected by Low-Density Parity-Check (LDPC) to guarantee strict recoverability. Conversely, for emotional features, we employ Deep Joint Source-Channel Coding (JSCC) analog transmission to prevent irreversible quantization errors and the cliff effect. Additionally, we develop a prior-guided diffusion reconstruction module at the receiving end. This module leverages a structural prior network to align the decoded semantics, which then steers the reverse diffusion process conditioned on the recovered affective features. Extensive experiments under both AWGN and Rayleigh fading channels demonstrate that HASCom significantly outperforms state-of-the-art baselines. Specifically, it achieves superior objective semantic similarity and subjective Mean Opinion Score (MOS) at low Signal-to-Noise Ratios (SNRs), while the JSCC transmission modules maintain an ultra-low inference latency of less than 0.1 ms, validating its high efficiency and robustness for practical deployments.

## 1. Introduction

Driven by the rapid proliferation of next-generation wireless networks (e.g., 6G) and the exponential growth of sensing devices, the communication paradigm is shifting from conventional bit-level transmission toward intelligent, semantic-aware interactions [1,2]. Traditional communication systems, which prioritize the exact recovery of raw bit sequences, face severe bandwidth bottlenecks when accommodating the massive data demands of smart devices [3,4]. To alleviate this, Semantic Communication (SemCom) has emerged as a transformative solution, focusing on extracting and transmitting only the essential meaning of the underlying data rather than the raw bitstream [5,6,7]. Furthermore, recent advancements in Generative Artificial Intelligence (GAI) have significantly reshaped the SemCom landscape, enabling receivers to reconstruct high-fidelity data from highly compressed semantic representations [8,9]. This evolution is particularly crucial for emerging paradigms such as Industry 5.0 and the Social Internet of Things (SIoT), which demand human-centric, emotion-aware, and highly reliable technological interfaces [10].

As a primary modality for social interaction, speech communication conveys not only the linguistic content (what is said) but also the rich affective state of the speaker (how it is said) [11,12,13]. However, most existing speech SemCom frameworks predominantly prioritize textual accuracy while neglecting the emotional tone, which is indispensable for genuine intent comprehension [14,15]. Neuroscience indicates that human cognition relies on the simultaneous processing of both linguistic and paralinguistic cues [16]. Therefore, there is an urgent need for a communication system capable of preserving both precise semantic meaning and profound emotional depth.

While foundational works like DeepSC [9] and its speech extensions (e.g., DeepSC-S [17], semantic-preserved speech transmission [14]) have successfully utilized Joint Source-Channel Coding (JSCC) to map speech signals into latent vectors, they primarily target content recovery. Recent studies have expanded this domain, exploring various efficiency and robustness enhancements in wireless audio semantics [11,18]. To address the loss of paralinguistic features, frameworks like EESC-S  [19] have successfully integrated emotion recognition into speech SemCom. Concurrently, to overcome the overly smoothed spectrograms typically generated by Mean Square Error (MSE) optimized decoders [15], recent literature has introduced diffusion probabilistic models to audio SemCom, notably the pioneering work by Grassucci et al. [20].

Despite these significant advancements, existing methods generally rely on homogeneous transmission strategies—concatenating discrete semantic and continuous emotional features into a shared analog space—or employ loosely conditioned generative processes. This uniform approach overlooks the distinct topological characteristics of linguistic and affective information. Consequently, it can lead to suboptimal bandwidth utilization and leaves the highly sensitive semantic content vulnerable to channel noise. Under severe fading conditions, such vulnerabilities may cause the reverse diffusion trajectory to diverge, occasionally resulting in semantic hallucinations and unnatural artifacts.

To comprehensively close the technical gaps left by EESC-S and Grassucci et al. [20], we propose the Heterogeneous Affective Speech Semantic Communication (HASCom) framework. In stark contrast to prior homogeneous architectures, HASCom innovatively decouples the speech signal into two distinct streams. It transmits discrete phoneme-level linguistic features via a highly reliable LDPC-protected digital link to guarantee absolute semantic integrity, while transmitting continuous affective embeddings through a Deep JSCC analog path to avoid irreversible quantization errors. At the receiver, rather than feeding vulnerable noisy representations directly into a denoiser, HASCom introduces a novel Structural Prior Network. This network utilizes the error-free digital semantic features to construct a robust monotonic alignment prior. This clean prior rigorously anchors the trajectory of the conditional diffusion model, eliminating channel-induced semantic hallucinations while successfully reconstructing expressive, emotion-rich Mel-spectrograms.

The main contributions of this study are summarized as follows:

- Heterogeneous Dual-Stream Architecture: We propose HASCom, a novel heterogeneous speech semantic communication framework that overcomes the bandwidth inefficiencies and cliff-effects inherent in existing homogeneous SemCom systems. By decoupling the speech signal, we enable a hybrid transmission capability: discrete phoneme-level linguistic features are protected by an LDPC-coded digital link to guarantee strict semantic recoverability, while continuous affective embeddings utilize Deep JSCC analog transmission to preserve fine-grained emotional details against dynamic channel noise.
- Prior-Guided Diffusion Reconstruction: We design a Structural Prior Network coupled with a conditional diffusion module to resolve the vulnerability of generative SemCom to channel impairments. Instead of feeding corrupted analog signals directly into the denoiser, this module leverages the robustly decoded digital semantics to construct an error-free monotonic alignment prior. This strategically anchors the reverse diffusion trajectory, effectively minimizing channel-induced semantic hallucinations and reconstruction errors, thereby synthesizing high-fidelity expressive speech under severe fading.
- Comprehensive Evaluation and Measurable Gains: We formulate an end-to-end optimization objective and rigorously evaluate the system under both AWGN and realistic Rayleigh multipath fading channels. The results demonstrate that HASCom achieves measurable and significant performance gains over state-of-the-art DeepSC-based baselines. It delivers superior objective cosine similarity and subjective Mean Opinion Scores (MOS) in low-SNR regimes, while the JSCC transmission link maintains an ultra-low inference latency of less than 0.1 ms, validating its high efficiency for practical deployment.

The remainder of this paper is organized as follows. Section 2 reviews the relevant literature and background work. Section 3 presents the overall system model and the high-level operational flow of the proposed HASCom framework. Section 4 formally defines the problem description, system assumptions, and the end-to-end optimization objectives. Section 5 details the specific network architecture, including the heterogeneous feature encoding, deep analog channel transmission, and the prior-guided diffusion reconstruction module. The simulation results and computational complexity analyses are presented in Section 6. Finally, Section 7 concludes the paper, acknowledges current limitations, and discusses future research directions.

## 2. Literature Review and Background Work

In this section, we review the prior literature most relevant to our proposed framework, categorizing them into speech semantic communication, emotion-aware frameworks, and diffusion-based reconstruction approaches. To explicitly position our contribution, Table 1 provides a structured comparison between HASCom and the closest competing works.

### 2.1. Speech Semantic Communication Systems

Following the paradigm-shifting success of DeepSC for text transmission [9], semantic communication (SemCom) was rapidly extended to the audio and speech domains. Weng et al. proposed DeepSC-S [17], leveraging Joint Source-Channel Coding (JSCC) to map speech signals directly into continuous latent vectors for analog transmission, significantly outperforming traditional separate source-channel coding mechanisms at low Signal-to-Noise Ratios (SNRs). Subsequently, Han et al. [14] developed a semantic-preserved communication system aimed at highly efficient speech transmission, while more recent studies by Xiao et al. [18] and Yeo et al. [11] explored deep wireless speech transmission over non-linear channels and enhanced extraction schemes. Limitation: These conventional speech SemCom systems predominantly focus on preserving the linguistic content (transcription accuracy) or treating speech as a generic analog signal, thereby discarding the rich paralinguistic and affective tone inherent in human voices.

### 2.2. Emotion-Aware Semantic Communication

Recognizing the necessity of paralinguistic cues for genuine human-centric interactions, recent efforts have attempted to integrate emotion recognition into the SemCom paradigm. The most notable work in this domain is the EESC-S framework proposed by Tan et al. [19], which extracts both semantic and emotional features to enhance reconstruction quality. Limitation: EESC-S employs a homogeneous transmission strategy, wherein discrete semantic data and continuous emotional features are simply concatenated into a shared latent space and transmitted via a uniform analog path. This homogeneous treatment ignores the fundamental topological differences between strict linguistic skeletons and flexible affective states, leading to suboptimal bandwidth utilization and leaving the highly sensitive semantic content vulnerable to channel fading.

### 2.3. Diffusion-Based Audio Reconstruction

Traditional SemCom receivers typically rely on Mean Square Error (MSE) optimized decoders, which are notorious for generating overly smoothed spectrograms with muffled auditory quality [15]. To address this, generative Artificial Intelligence, particularly diffusion probabilistic models [21], has been introduced. A pioneering effort by Grassucci et al. [20] applied diffusion models to wireless audio semantic communication, demonstrating unprecedented generative fidelity. Limitation: Directly applying diffusion models over noisy wireless channels introduces a critical vulnerability. Unconditional or loosely conditioned reverse diffusion processes are highly susceptible to channel impairments. If the guiding semantic latent vector is corrupted by multi-path fading or additive noise, the diffusion trajectory severely diverges, leading to severe semantic hallucinations (e.g., synthesizing incorrect phonemes) and artificial distortions.

### 2.4. Summary of Technical Increments

In stark contrast to the aforementioned works, our HASCom framework closes these technical gaps through two core innovations. First, it abandons the homogeneous transmission paradigm in favor of a heterogeneous dual-stream architecture, routing strict semantic features through an error-free digital link and continuous emotional features through an analog JSCC link. Second, it resolves the vulnerability of diffusion-based receivers by introducing a Structural Prior Network, which utilizes the robustly decoded digital semantics to firmly anchor the generative diffusion trajectory, eliminating hallucinations even under severe Rayleigh fading.

## 3. System Model

In this section, we propose the Heterogeneous Affective Speech Semantic Communication (HASCom) system, which establishes a novel dual-stream transmission architecture. By decoupling the speech signal into a discrete semantic stream and a continuous affective stream, the system balances linguistic accuracy with acoustic fidelity. Unlike prior homogeneous architectures that process all latent representations uniformly, HASCom ensures that the reconstructed speech maintains rigorous semantic intelligibility via robust digital coding, while simultaneously exhibiting rich emotional fidelity preserved through deep analog transmission.

**Figure 1:**
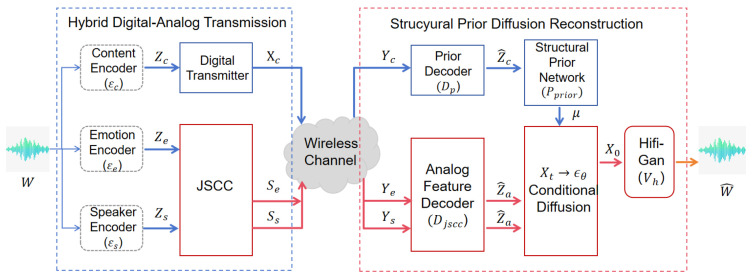
Overall architecture of the proposed Heterogeneous Affective Speech Semantic Communication System (HASCom). The framework decouples speech into a discrete semantic stream and a continuous affective stream for heterogeneous transmission. At the receiver, the structural prior network establishes semantic alignment, while the recovered affective features guide the diffusion model to synthesize high-fidelity speech with precise intelligibility and robust emotional expressiveness.

As illustrated in Figure 1, HASCom has two core stages: the Heterogeneous Semantic-Affective Transmission module and the Prior-Guided Affective Diffusion Reconstruction module. This transmission module use a hybrid transmission method. It uses a reliable digital channel to transmit the text content ($Z_{c}$), ensuring the complete preservation of semantics. Simultaneously, the analog JSCC encoder is used to directly map the emotions ($Z_{e},Z_{s}$) directly into continuous channel symbols, thereby reducing the loss of subtle voice intonation changes. At the receiving end, the reconstruction module adopts a generation mode of text prior followed by diffusion. The structure prior network obtains (μ) and the Conditional Diffusion model processes the prior based on the recovered simulated affective cues (${\hat{Z}}_{a}$), so that it can get high-fidelity speech reconstruction resilient to channel impairments.

### 3.1. Hybrid Digital-Analog Transmission

Let $W\in R^{L}$ denote the raw input speech waveform of length *L*. The system first decomposes *W* into disentangled latent representations corresponding to linguistic content, emotional state, and speaker identity. Multi-modal feature extraction is formulated as

(1) $$Z_{c}=E_{c}\left(W;\omega_{c}\right),\;Z_{e}=E_{e}\left(W;\omega_{e}\right),\;Z_{s}=E_{s}\left(W;\omega_{s}\right),$$

where $E_{c}$, $E_{e}$, and $E_{s}$ denote the content, emotion, and speaker encoders parameterized by $\omega_{c}$, $\omega_{e}$, and $\omega_{s}$, respectively. Here, $Z_{c}\in R^{D_{c}}$ represents the discrete phoneme-level linguistic features, while $Z_{e}\in R^{D_{e}}$ and $Z_{s}\in R^{D_{s}}$ represent the continuous embedding vectors for emotion and speaker timbre.

#### 3.1.1. Discrete Semantic Link

To reduce semantic errors caused by channel noise, the language feature $Z_{c}$ is transmitted through the traditional digital channel. The features are first quantized and source-encoded into a binary bit sequence, then undergo channel encoding and digital modulation. This process generates discrete digital transmission symbols $X_{c}$:

(2) $$X_{c}=M\left(C\left(Q\left(Z_{c}\right)\right)\right)\in C^{N_{c}},$$

where Q(·) denotes the quantization operation, C(·) represents the channel coding, M(·) is the digital modulation function, and $N_{c}$ denotes the frame length of the digital content symbols.

#### 3.1.2. Continuous Affective Link

For the speaker’s tone and emotions, we use analog transmission. To transmit multiple vectors through the channel, we concatenate the emotion vector $Z_{e}$ and the speaker vector $Z_{s}$ to form a unified expressive style vector $Z_{a}$. Then, this vector is mapped directly to continuous-valued analog channel symbols $S_{a}$ through the deep Joint Source-Channel Coding (JSCC) encoder $E_{jscc}$:

(3) $$S_{a}=E_{jscc}\left(Z_{a};\omega_{j}\right)\in C^{N_{a}},$$

where $\omega_{j}$ denotes the parameters of the JSCC encoder, and $N_{a}$ represents the dimension of the analog affective symbols. This JSCC-based analog transmission preserves fine-grained emotional features more effectively than traditional digital coding, which suffers from irreversible quantization distortion.

The encoded symbols $X_{c}$ and $S_{a}$ are transmitted through a wireless channel. To accurately simulate the digital channel and the JSCC channel, we defined the received signals $Y_{c}$ and $Y_{a}$ separately:

(4) $$\begin{array}{cc} Y_{c} & =h_{c}X_{c}+n_{c},\\ Y_{a} & =h_{a}S_{a}+n_{a}, \end{array}$$

where $h_{c},h_{a}$ represent the digital channel and the jscc channel respectively, and $n_{c},n_{a}∼\mathrm{CN}\left(0,\sigma^{2}I\right)$ denote the additive white Gaussian noise (AWGN). The receiver employs a heterogeneous decoding strategy to recover the latent features from these two distinct signal streams.

#### 3.1.3. Robust Semantic Restoration

For the digital signal $Y_{c}$, the receiving process mainly aims to restore the correct text of the speech. We do not only consider semantic restoration as a strict bit decoding, but rather see it as a structural decoding process. We have defined a prior decoder named $D_{p}$ to turn the noisy digital signals back into clear word information:

(5) $${\hat{Z}}_{c}=D_{p}\left(Y_{c};\psi_{c}\right),$$

where $\psi_{c}$ represents the parameters of the decoder.

Through the reliable digital link, the prior decoder $D_{p}$ effectively recovers the discrete semantic representations. Rather than completely eliminating all physical noise, this decoder minimizes the semantic reconstruction error under dynamic channel conditions, ensuring that the output ${\hat{Z}}_{c}$ preserves the original linguistic structure and phonetic meaning. By substantially mitigating channel-induced distortions, it provides a robust and reliable foundation for the subsequent generative diffusion process.

#### 3.1.4. Affective Feature Reconstruction via Neural Inversion

The continuous signal flow $Y_{a}$ takes information about emotions and the speaker, which requires a decoding capability that is adaptive to channel variations. We employed a specialized deep neural feature decoder, denoted as $D_{jscc}$, to handle this analog signal. It can extract expressive latent vectors from noisy channel symbols:

(6) $${\hat{Z}}_{a}=D_{jscc}\left(Y_{a};\psi_{j}\right),$$

where $\psi_{j}$ represents the trainable parameters of the decoder. This network is trained to implicitly perform joint channel equalization and source denoising, effectively filtering out channel noise $n_{a}$ and preserving the fine-grained values of the emotion and speaker embeddings. The recovered ${\hat{Z}}_{a}$ is then divided into emotion estimate ${\hat{Z}}_{e}$ and speaker estimate ${\hat{Z}}_{s}$, which will be the conditions for the subsequent diffusion generation.

#### 3.1.5. Structural Prior Generation

The recovery process is a process of obtaining priors from text. We employed a structured prior network named $P_{prior}$, which has a text encoder and a monotonic alignment search (MAS) mechanism. With the text feature ${\hat{Z}}_{c}$ as input, it predicts the mean μ of the terminal Gaussian distribution for the diffusion process:

(7) $$\mu =P_{prior}\left({\hat{Z}}_{c};\psi_{p}\right)\in R^{M\times T},$$

where *M* is the number of Mel-frequency bands and *T* is the temporal duration. Here, μ provides the structural alignment of the speech content, serving as the foundation for the subsequent diffusion process.

#### 3.1.6. Affective-Guided Reverse Diffusion

The conditional diffusion generator, denoted as $G_{d}$, uses the obtained ${\hat{Z}}_{a}$ as the main conditional input to adjust the reverse denoising trajectory. Unlike unconditional generation, our network can better complete denoising through prior conditions. The prediction of the noise component $\epsilon_{\theta }$ at each diffusion time step *t* is mathematically formulated as

(8) $$\epsilon_{\theta }=G_{d}\left(X_{t},\mu ,t,{\hat{Z}}_{a};\psi_{d}\right)\in R^{M\times F},$$

where $\psi_{d}$ represents the learnable parameters of the neural generator, and $X_{t}$ denotes the noisy Mel-spectrogram state at time *t*.

In this formulation, the recovered vector ${\hat{Z}}_{a}$ will be projected and injected into the denoising network. This ensures that the generated speech $X_{0}$ retains the complex intonation and timbre passed through jscc, while also adhering to the strict language alignment constraints defined by the semantic structure prior μ. The prior μ provides the restoration framework, and ${\hat{Z}}_{a}$ enables the restored speech to exhibit more emotion, thereby facilitating collaborative reconstruction of the target speech.

Finally, the denoised Mel-spectrogram $X_{0}$, obtained at the end of the reverse diffusion process, is transformed into the time-domain waveform to complete the end-to-end communication loop. We employ a pretrained HiFi-GAN vocoder, denoted as $V_{h}$, to synthesize the high-fidelity waveform $\hat{W}$ from the spectrogram:

(9) $$\hat{W}=V_{h}\left(X_{0}\right)\in R^{L}.$$

This non-autoregressive vocoding stage ensures that the final output preserves the perceptual quality and phase continuity of the original speech signal.

## 4. Problem Description and Formulation

Before introducing the architectural details, this section formally defines the signal model, explicitly states the communication channel assumptions, and outlines the unified end-to-end optimization objective.

### 4.1. Signal and Channel Assumptions

Let $W\in R^{T}$ denote the raw input speech waveform. The proposed HASCom framework decouples *W* into a discrete semantic representation and a continuous affective representation, which are transmitted over a heterogeneous dual-stream link (digital and analog).

To evaluate the system under unified and realistic wireless conditions, let $X\in C^{N}$ represent the complex-valued transmitted symbols for either stream. The received signal *Y* is mathematically modeled as

(10) Y=hX+n,

where $n∼\mathrm{CN}(0,\sigma^{2}I)$ represents the Additive White Gaussian Noise (AWGN). The channel fading coefficient *h* is explicitly defined according to the deployment scenario:

- AWGN Channel: To simulate an ideal line-of-sight environment, h=1.
- Rayleigh Fading Channel: To simulate complex multipath propagation, *h* is modeled as independent and identically distributed (i.i.d.) complex Gaussian random variables, i.e., h∼CN(0,1).

This unified definition resolves any ambiguity and applies consistently to all subsequent theoretical derivations and experimental setups.

### 4.2. End-to-End Optimization Objective

Let θ denote the comprehensive set of learnable parameters across the entire end-to-end transceiver architecture. The overarching communication objective is to reconstruct a high-fidelity speech waveform $\hat{W}$ by minimizing the expected end-to-end distortion over the stochastic channel conditions. The optimization problem is formulated as

(11) $$\min_{\theta }E_{W,h,n}\left[L(W,\hat{W})\right].$$

To effectively guide model convergence across multiple semantic and physical modalities, the composite loss function L is formulated as

(12) $$L=L_{d}+\lambda_{a}L_{a}+\lambda_{s}\left(L_{s}+L_{t}\right),$$

where $L_{d}$, $L_{a}$, $L_{s}$, and $L_{t}$ represent the diffusion denoising, analog consistency, semantic alignment, and temporal duration losses, respectively. The weights $\lambda_{a}$ and $\lambda_{s}$ are unified predefined hyperparameters that balance the trade-off between generative quality, transmission robustness, and structural alignment. The specific formulations of these neural network modules and corresponding loss components will be detailed in the subsequent system model sections. For ease of reference, a comprehensive summary of the key mathematical notations used throughout this paper is provided in Table 2.

## 5. Heterogeneous Affective Speech Semantic Communication System

In this section, we provide a comprehensive description of the proposed Heterogeneous Affective Speech Semantic Communication System (HASCom). As depicted in Figure 1, HASCom establishes a new communication mode. The traditional framework either treats all speech features equally or relies solely on digital encoding, while HASCom decomposes speech into semantic and emotional parts and transmits them through traditional digital channels and jscc respectively. This architecture can ensure the reconstructed speech maintains semantic accuracy and rich emotional fidelity. In the following subsections, we will detail its core components: the heterogeneous semantic emotional transmission module, the emotion diffusion reconstruction module based on prior knowledge, and the corresponding two-stage optimization strategy.

### 5.1. Heterogeneous Semantic-Affective Feature Encoding

To ensure the text content is accurately conveyed while retaining rich emotional expression details, we proposed a heterogeneous semantic emotional feature-encoding mechanism at the sending end. Unlike the traditional method of integrating all features into a unified embedding for unified transmission, this mechanism strategically divides the encoding process into two parts based on the physical properties of the features, generating a robust digital transmission for semantics and an analog transmission for emotions.

To achieve heterogeneous transmission mode, our system splits the original voice waveform *W* into two vectors. We have designed specific extraction pathways that map the high-dimensional temporal signal into compact semantic and affective embeddings, utilizing state-of-the-art self-supervised encoders as the backbone feature extractors.

#### 5.1.1. Semantic Content Extraction

To ensure text accuracy and avoid bias from the speaker’s identity and emotional tone, we adopted the Wav2Vec 2.0 framework [22] as the core content encoder. This module acts as a language filter, processing the original waveform *W* and extracting a series of frame-level discrete representations. Different from traditional spectral features, these deep latent variables can catch the context structure of the text and have strong robustness to channel changes. From a mathematical perspective, the semantic extraction process maps the time-domain signal to a language feature space through the encoder function $E_{c}$, expressed as

(13) $$Z_{c}=E_{c}\left(W;\omega_{c}\right)\in R^{T_{c}\times D_{c}},$$

where $\omega_{c}$ denotes the pre-trained parameters, $T_{c}$ represents the effective temporal resolution aligned with phonemic boundaries, and $D_{c}$ is the feature dimension. These representations $Z_{c}$ make the semantic foundation, providing the solid structural foundation for the subsequent digital transmission.

#### 5.1.2. Unified Affective Extraction

To obtain the speaker’s voice characteristics and emotional features, we proposed a unified emotion extraction strategy based on the WavLM architecture [23]. We do not deliberately distinguish emotions from the speaker; instead, we combine them into a single vector.

This extraction mechanism combines the extracted emotions and speaker features into a unified input suitable for simulating transmission. While ensuring that the dimensions do not become disordered, we retain complete information about the emotion intensity and the speaker’s identity. We construct the unified affective latent $Z_{a}$ via a concatenation operation:

(14) $$Z_{a}=\left[Z_{e}⊕Z_{s}\right]\in R^{D_{e}+D_{s}},$$

where ⊕ denotes the vector concatenation operator, and $Z_{e}\in R^{D_{e}}$ and $Z_{s}\in R^{D_{s}}$ are the extracted emotion and speaker embeddings. This composite vector $Z_{a}$ will be fed into the JSCC encoder, ensuring that the continuous channel symbols can simultaneously contain the general intonation and timbre with high fidelity.

### 5.2. Channel Transmission Model

The HASCom framework operates in a hybrid wireless environment where semantics and emotions are transmitted through different channels. To comprehensively evaluate the system’s robustness in practical scenarios, we have modeled these two wireless transmission scenarios under both the standard additive white Gaussian noise (AWGN) channel and the more complex Rayleigh fading channel.

#### 5.2.1. Digital Semantic Transmission

To guarantee the rigorous recoverability of the linguistic skeleton, the quantized semantic features are transmitted via a classic digital link protected by Low-Density Parity-Check (LDPC) codes. While our baseline design assumes standard LDPC performance, practical communication scenarios often face severe bit distortion caused by complex channel interference. To mitigate this limitation, advanced iterative decoding schemes can be integrated in the receiver. For instance, the recently proposed Bit-Interleaved Coded Modulation with Iterative Decoding (BILCM-ID) system [24] provides a highly advanced solution by utilizing an improved stopping criterion, which effectively resolves practical bit distortions while maintaining superior computational efficiency. The received digital signal $Y_{c}$ is modeled as

(15) $$Y_{c}=h_{c}S_{c}+n_{c},$$

where $S_{c}\in C^{N_{c}}$ denote the QAM-modulated symbols derived from the LDPC-coded bits, and $n_{c}∼\mathrm{CN}\left(0,\sigma_{c}^{2}I\right)$ is the Gaussian noise vector. To accommodate different wireless environments, the channel coefficient $h_{c}$ is set to 1 for the ideal AWGN channel, and $h_{c}∼\mathrm{CN}\left(0,1\right)$ for the Rayleigh fading channel to simulate complex multipath propagation.

#### 5.2.2. Deep Analog Affective Transmission (JSCC)

Unlike the discrete semantic stream, the affective features $Z_{a}$ (extracted by the large-scale WavLM backbone) represent continuous-valued deep representations where the Euclidean distance directly correlates with perceptual similarity. Traditional digital coding would introduce irreversible quantization noise and suffer from the cliff effect when channel conditions deteriorate. Therefore, we employ a Deep JSCC strategy.

The JSCC encoder $E_{jscc}$ functions as a neural projection network, mapping the high-dimensional affective latent $Z_{a}$ directly into a sequence of complex-valued channel input symbols. To ensure strict adherence to the hardware transmission power constraints and to maximize energy efficiency, we impose a global power normalization layer immediately following the neural mapping. The transmitted analog symbols $S_{a}$ are obtained by

(16) $${\tilde{S}}_{a}=E_{jscc}\left(Z_{a};\omega_{j}\right),\;S_{a}=\sqrt{P}\frac{{\tilde{S}}_{a}}{\sqrt{E[∥{\tilde{S}}_{a}{∥}^{2}]}},$$

where *P* is the average transmission power constraint, and the normalization ensures $E[∥S_{a}{∥}^{2}]=P$. This operation aligns the variance of the deep features with the physical channel capacity, preventing signal saturation or attenuation.

After propagating through the wireless channel, the received analog signal is expressed as

(17) $$Y_{a}=h_{a}S_{a}+n_{a},$$

where $h_{a}$ and $n_{a}$ denote the fading and noise components for the analog link. To counteract the phase shifts and amplitude attenuations introduced by the Rayleigh fading scenario, a Zero-Forcing (ZF) equalizer is applied at the receiver under the assumption of perfect Channel State Information (CSI), yielding the equalized signal ${\hat{Y}}_{a}=Y_{a}/h_{a}$. Subsequently, the neural decoder $D_{jscc}$ acts as a non-linear Minimum Mean Square Error (MMSE) estimator.

In this paradigm, the system learns to allocate essential emotional representations into the more robust sub-components of the channel. This leads to graceful degradation: even if the noise is heavy, it only causes subtle variations in emotional expressiveness rather than a catastrophic communication failure.

### 5.3. Diffusion Model

#### 5.3.1. Theoretical Foundation

We adapt the score-based generative framework to facilitate robust speech reconstruction. The traditional conditional diffusion model follows a backward process starting from the standard isotropic Gaussian distribution N(0,I), our approach use the semantics recovered from the digital channel to construct a structural prior. Specifically, we use the semantic skeleton to define the mean μ of the terminal distribution. This strategy provides a valuable starting point for the generation process, enabling the model to focus on refining the fine acoustic details and emotional intonation rather than synthesizing language structures from pure noise.

We do not model the forward degradation process as a transformation into random noise, but rather regard it as a mean-reverting Ornstein–Uhlenbeck (OU) stochastic differential equation. Given the ground-truth Mel-spectrogram $X_{0}$, the state evolution at time *t* is governed by

(18) $$dX_{t}=\frac{1}{2}\beta_{t}\left(\mu -X_{t}\right)dt+\sqrt{\beta_{t}}dw_{t},\;t\in \left[0,T\right],$$

where $\beta_{t}$ represents the non-negative noise schedule and $w_{t}$ denotes the standard Wiener process. The μ is the structural prior, and it is deterministically predicted from the decoded semantic features ${\hat{Z}}_{c}$ via the Structural Prior Network. Under this formulation, when *t* approaches *T*, the distribution of the noisy state $X_{T}$ converges to N(μ,I). This means that the final distribution of the forward process is determined by the language content rather than by the standard Gaussian distribution.

The generation of high-fidelity speech is equal to the reverse-time solution of the SDE. To ensure the reconstruction possesses the correct emotional intensity and speaker timbre, we formulate the reverse dynamics as a probability flow Ordinary Differential Equation (ODE) conditioned on the analog affective cues. The deterministic trajectory evolving from the structural prior $X_{T}$ back to the data distribution $X_{0}$ is defined as

(19) $$dX_{t}=\frac{1}{2}\beta_{t}\left[\left(\mu -X_{t}\right)-\epsilon_{\theta }\left(X_{t},\mu ,t,{\hat{Z}}_{a}\right)\right]dt,$$

where $\epsilon_{\theta }\left(X_{t},\mu ,t,{\hat{Z}}_{a}\right)$ approximates the score function $\nabla_{X_{t}}\log p_{t}\left(X_{t}|{\hat{Z}}_{a}\right)$.

In this theoretical framework, the term $\left(\mu -X_{t}\right)$ acts as a conservative force field, fixing the generated trajectory based on the prior knowledge, thereby ensuring the comprehensibility of the language. Additionally, the neural score estimator $\epsilon_{\theta }$, conditioned on the recovered emotional features ${\hat{Z}}_{a}$, introduces the necessary gradient field to synthesize fine nuances in tone and timbre. Through this approach, we construct our diffusion model.

#### 5.3.2. Structural Prior Network

The structural prior network first needs to obtain the semantic prior, which defines the final state of the proposed shortcut diffusion trajectory. To transform the non-isochronous linguistic features ${\hat{Z}}_{c}$ into the time-synchronized acoustic skeleton μ, we formulate the alignment process as a latent variable optimization problem solved via Monotonic Alignment Search (MAS).

The Structural Prior Network $P_{prior}$ will first project the digital semantic inputs into a sequence of unaligned distribution parameters $\tilde{\mu }$. The MAS algorithm then identifies the optimal monotonic path $A^{*}$ that maximizes the log-likelihood of the ground-truth spectrogram $X_{0}$ being generated from this prior distribution. This process aligns the discrete phoneme frames to the continuous acoustic time axis, yielding the aligned mean:

(20) $$\mu =\mathrm{Align}(\tilde{\mu },A^{*}).$$

We train this aligned mean to serve as the geometric center of the diffusion terminal distribution, postulating that $X_{T}∼N\left(\mu ,I\right)$. So, the main optimization objective is to minimize the Euclidean distance between the generated prior and the true value, and its form is the structural alignment loss:

(21) $$L_{s}={∥\mu -X_{0}∥}_{2}^{2}.$$

We want to ensure the system can autonomously determine the temporal structure during inference without ground-truth guidance, so we jointly optimize an internal duration predictor to approximate the optimal token durations. The target durations $d_{mas}$ are derived by aggregating the alignment path $A^{*}$ along the time axis. We impose the Temporal Duration Loss on the predicted durations $d_{pred}$ in the logarithmic domain to robustly handle the dynamic range of speech rhythm:

(22) $$L_{t}={∥\log\left(d_{pred}\right)-\log\left(d_{mas}\right)∥}_{2}^{2}.$$

By minimizing this objective, the network learns to construct a stable, time-aligned acoustic skeleton μ solely from the digital linguistic stream, providing the necessary deterministic boundary condition for the subsequent affective diffusion process. Inference-Time Alignment Generation: While the Monotonic Alignment Search (MAS) algorithm provides the optimal alignment path $A^{*}$ during training by leveraging the ground-truth spectrogram $X_{0}$, this acoustic target is fundamentally unavailable during the inference phase at the receiver. To bridge this gap, the trained internal duration predictor entirely replaces the MAS module. Specifically, during inference, the duration predictor processes the unaligned discrete semantic features to estimate a continuous duration value $d_{pred}$ for each linguistic token. To map these abstract tokens to the discrete acoustic time axis, these continuous predictions are rounded to the nearest integer, dictating the exact number of acoustic frames each semantic token will occupy. The unaligned semantic vectors $\tilde{\mu }$ are then deterministically duplicated (expanded) along the time axis according to these integer durations. This autonomous expansion constructs the fully time-synchronized structural prior μ without requiring any external ground-truth acoustic reference, thereby enabling independent, end-to-end speech reconstruction at the receiver.

#### 5.3.3. Affective-Guided Diffusion Optimization

To reconstruct semantically faithful and emotionally expressive speech, we propose an emotion-conditioned diffusion architecture that explicitly embeds the recovered affective features as dominant guidance signals in the reverse generative process. The core of this module is the neural score estimator $\epsilon_{\theta }$, implemented as an improved U-Net architecture tailored to synergize the rigid semantic skeleton with the emotion.

To strictly enforce the linguistic alignment constraints, we adopt a structural concatenation strategy at the network input. The prior μ, derived from the prior network, is concatenated to the noisy intermediate variable $X_{t}$ along the channel dimension. This combined tensor serves as the diffusion input, defines the generated search space, and enables the network to focus its capabilities on refining acoustic textures rather than inferring the geometric structure from scratch.

Crucially, to embed the continuous emotional elements efficiently within the standard U-Net backbone, we propose a highly effective multi-level Feature-wise Linear Modulation (FiLM) mechanism. Instead of relying entirely on complex attention modules that incur heavy computational overhead, we utilize direct feature modulation. Let $x_{l}\in R^{B\times C\times H\times W}$ denote the feature map at the *l*-th layer of the U-Net, and let $c\in R^{B\times D_{cond}}$ denote the injected affective condition (derived from the analog features ${\hat{Z}}_{a}$). The FiLM module employs a Multi-Layer Perceptron (MLP) with a Mish activation function to project the condition *c* into channel-wise scaling ($\gamma_{l}$) and shifting ($\beta_{l}$) parameters:

(23) $$\left[\gamma_{l},\beta_{l}\right]=\mathrm{MLP}\left(c\right)\in R^{B\times 2C},$$

where both $\gamma_{l}$ and $\beta_{l}$ are subsequently reshaped to $R^{B\times C\times 1\times 1}$ to align with the spatial dimensions of the feature map. The modulated output is then obtained via a channel-wise affine transformation:

(24) $$\mathrm{FiLM}\left(x_{l},c\right)=\gamma_{l}\circ x_{l}+\beta_{l},$$

where ∘ denotes element-wise multiplication broadcasted across the spatial dimensions. By injecting this FiLM modulation into the residual blocks of the U-Net, the system ensures that the speaker’s voice characteristics and emotional intensity are deeply and uniformly integrated into the generation process across multiple resolution scales.

With the neural estimator parameterized and structurally optimized, the generative reconstruction corresponds to solving the reverse-time probability flow Ordinary Differential Equation (ODE). Starting from the terminal distribution centered on the semantic prior, this trajectory develops in a deterministic manner towards the distribution area of pure data according to the gradient estimation rule of the network:

(25) $$dX_{t}=\frac{1}{2}\beta_{t}\left[\left(\mu -X_{t}\right)-\epsilon_{\theta }\left(X_{t},\mu ,t,{\hat{Z}}_{a}\right)\right]dt,$$

where $\beta_{t}$ sets the pace of the process. In this dynamic equation, the drift term $\left(\mu -X_{t}\right)$ acts as a structural constraint that preserves linguistic intelligibility. Concurrently, the neural score estimator $\epsilon_{\theta }\left(\cdot \right)$ introduces the necessary gradient corrections to synthesize the fine-grained tonal and timbral details, explicitly conditioned on the recovered affective features.

The optimization objective is to train the network parameters θ to accurately approximate this score function. Aligned with the theoretical formulation of the mean-reverting stochastic differential equation, we formulate the diffusion denoising loss $L_{d}$ as the weighted expected mean squared error between the predicted noise term and the actual Gaussian noise ξ injected during the forward process:

(26) $$L_{d}=E_{t,X_{0},\xi }\left[\lambda_{t}{∥\epsilon_{\theta }\left(X_{t},\mu ,t,{\hat{Z}}_{a}\right)-\xi ∥}_{2}^{2}\right],$$

where *t* is sampled uniformly from the time horizon [0,T], $X_{t}$ is sampled from the forward transition kernel, and $\lambda_{t}$ denotes the weighting coefficient. By minimizing $L_{d}$, the model learns to effectively utilize the analog affective cues ${\hat{Z}}_{a}$ to reconstruct the missing emotional details upon the semantic prior framework.

### 5.4. Training Strategy

#### 5.4.1. Analog Affective Transmission Optimization

The training process of HASCom is divided into two independent stages to ensure its stability. The first stage focuses on establishing a robust analog channel for the affective features. The detailed optimization procedure is outlined in Algorithm 1.

#### 5.4.2. Generative Reconstruction Optimization

The focus of the next stage lies in the joint optimization of the structural prior and the emotional diffusion model. In this stage, the JSCC module acts as a fixed and noise-resistant feature extractor.

Before detailing the algorithmic procedure, we formally define the affective encoding operations referenced in the pseudocode. Let $E_{a}$ denote the composite affective encoder backbone (comprising both the emotion and speaker extraction pathways). Given the input waveform *W*, the aggregation function Agg(·) operates by concatenating the discrete outputs of these pathways along the feature dimension to form the unified affective latent representation. Mathematically, this is expressed as $Z_{a}=\mathrm{Agg}\left(E_{a}\left(W\right)\right)=\left[E_{e}\left(W\right)⊕E_{s}\left(W\right)\right]$. The complete training process is detailed in Algorithm 2.
**Algorithm 1** Stage I: Training Procedure for the Analog Affective Link**Input:** **Frozen Modules:** Emotion Encoder $E_{e}$, Speaker Encoder $E_{s}$;
     **Trainable Modules:** JSCC Encoder $E_{jscc}$, JSCC Decoder $D_{jscc}$ (collectively $\theta_{jscc}$);
     **Hyperparameters:** Learning rate η, batch size *B*, transmit power *P*.
**Output:** Optimized Parameters $\theta_{jscc}$.
 1:**Initialize** trainable parameters $\theta_{jscc}$ randomly. 2:**repeat** 3:   **for** each batch W∼D (batch size *B*) **do** 4:        Extract emotion vectors: $Z_{e}=E_{e}\left(W\right)$ 5:        Extract speaker vectors: $Z_{s}=E_{s}\left(W\right)$ 6:        Concatenate latent features: $Z_{a}=\left[Z_{e}⊕Z_{s}\right]$ 7:        Map to channel symbols: ${\tilde{S}}_{a}=E_{jscc}\left(Z_{a}\right)$ 8:        Apply Power Normalization: 9:        $S_{a}=\sqrt{P}\cdot \frac{{\tilde{S}}_{a}}{\sqrt{E∥{\tilde{S}}_{a}{∥}^{2}+\epsilon }}$10:        Sample channel state $h_{a}∼\mathrm{CN}\left(0,1\right)$ and noise $n_{a}∼\mathrm{CN}\left(0,\sigma^{2}\right)$11:        Transmission: $Y_{a}=h_{a}\cdot S_{a}+n_{a}$12:        Neural Inversion: ${\hat{Z}}_{a}=D_{jscc}\left(Y_{a}\right)$13:        Compute Reconstruction Loss:14:        $L_{a}=\frac{1}{B}\sum_{i=1}^{B}{∥Z_{a}^{\left(i\right)}-{\hat{Z}}_{a}^{\left(i\right)}∥}_{2}^{2}$15:        Update gradients: $\theta_{jscc}\leftarrow \theta_{jscc}-\eta \nabla_{\theta }L_{a}$16:   **end for**17:**until** convergence or max epochs reached18:**return** $\theta_{jscc}$

### 5.5. End-to-End Training Protocol

We adopted the sequential optimization method and mimicked the physical causal relationship of the channel. This ensured that the generative receiver could effectively receive training to cope with the complexity of speech synthesis and the randomness of channel distortion.

The system is firstly trained in an ideal, noise-free environment to establish a robust generative baseline. The semantic quantization and Deep JSCC modules are bypassed, feeding the ground-truth linguistic features $Z_{c}$ and affective features $Z_{a}$ directly into the reconstruction module. The structural prior network and the conditional diffusion model are jointly optimized to learn the fundamental mapping from heterogeneous latent representations to high-fidelity Mel-spectrograms. This pre-training strategy ensures that the generative receiver accurately captures the intrinsic mapping between the linguistic structural prior and the fine-grained acoustic details, isolated from channel impairments. This mitigates the risk of training divergence and gradient instability caused by severe signal distortion during the initial optimization phase.

We introduced physical channel simulation based on stable generation for fine-tuning. We used deep jscc to inject random channel noise $n_{a}$ and fading $h_{a}$ into the affective stream to simulate real-world transmission conditions. Then, the pre-trained diffusion model was allowed to adapt to the distortion features ${\hat{Z}}_{a}$. In this stage, the model transitions from learning the fundamental generative mapping under clean conditions to adapting its reconstruction to channel-impaired inputs. It learns to compensate for the distortion patterns introduced by the physical channel while preserving the complementary information from both the digital semantic and analog affective streams.

The structural prior network processes the semantics and generates the prior μ, while the diffusion model utilizes the affective condition ${\hat{Z}}_{a}$ to refine this prior. Although the internal loss of the diffusion model is to calculated on the noise residual, the total optimization objective is equivalent to minimizing the reconstruction error between the generated spectrogram and the original ground truth. Our ultimate training goal is to minimize the divergence between the ground-truth spectrogram *S* (derived from *W*) and the reconstructed spectrogram $\hat{S}$ (equivalent to $X_{0}$):

(27) $$\min E\left[{∥S-\hat{S}∥}_{2}^{2}\right].$$

By minimizing this objective, the system can make the final output converges to the original spectral distribution, and at the same time, it can preserve both the linguistic content and the rich paralinguistic details.
**Algorithm 2** Stage II: Prior-Guided Affective Diffusion Optimization**Input: Frozen Modules:** JSCC Encoder/Decoder $\theta_{jscc}$, Semantic Encoder $E_{c}$;     **Trainable Modules:** Structural Prior Network $P_{prior}$ (including Duration Predictor),     Diffusion U-Net $\epsilon_{\theta }$;     **Data:** Speech dataset D, Ground-truth Mel-spectrogram $X_{0}$;
     **Hyperparameters:** Loss weights $\lambda_{a},\lambda_{s}$, noise schedule $\beta_{t}$.
**Output:** Optimized Generative Parameters $\theta_{gen}=\left\{P_{prior},\epsilon_{\theta }\right\}$.
 1:**Initialize** $\theta_{gen}$ randomly. 2:**repeat** 3:   **for** each batch $(W,X_{0})∼D$ **do** 4:     **Digital Stream:** Extract & Quantize semantic features: 5:        ${\hat{Z}}_{c}\leftarrow \mathrm{Quantize}\left(E_{c}\left(W\right)\right)$ 6:     **Analog Stream:** Transmit affective features via frozen JSCC: 7:        $Z_{a}=\mathrm{Agg}\left(E_{a}\left(W\right)\right)$ 8:        ${\hat{Z}}_{a}=D_{jscc}\left(\mathrm{Channel}\left(E_{jscc}\left(Z_{a}\right)\right)\right)$ 9:     Predict unaligned distributions: $\tilde{\mu }=P_{prior}\left({\hat{Z}}_{c}\right)$10:     Monotonic Alignment Search (MAS):11:        $A^{*}=\mathrm{MAS}\left(\tilde{\mu },X_{0}\right)$12:     Get aligned semantic skeleton & durations:13:        $\mu =\mathrm{Align}\left(\tilde{\mu },A^{*}\right),\;d_{mas}=\mathrm{Sum}\left(A^{*}\right)$14:     Compute Structural Losses:15:        $L_{s}=∥\mu -X_{0}{∥}^{2},\;L_{t}={∥\log d_{pred}-\log d_{mas}∥}^{2}$16:     Sample time t∼[0,T] and noise ξ∼N(0,I)17:     Sample noisy state $X_{t}$ via OU forward process anchored at μ:18:        $X_{t}=\rho_{t}X_{0}+\left(1-\rho_{t}\right)\mu +\delta_{t}\xi$19:     Predict noise residual with Affective FiLM conditioning:20:        $\hat{\xi }=\epsilon_{\theta }\left(X_{t},\mu ,t,{\hat{Z}}_{a}\right)$21:     Compute Diffusion Loss:22:        $L_{d}={∥\hat{\xi }-\xi ∥}^{2}$23:     Aggregate Total Loss:24:        $L_{total}=L_{d}+\lambda_{a}L_{a}+\lambda_{s}\left(L_{s}+L_{t}\right)$25:     Update gradients: $\theta_{gen}\leftarrow \theta_{gen}-\eta \nabla_{\theta }L_{total}$26:   **end for**27:**until** convergence28:**return** $\theta_{gen}$

## 6. Simulation

In this section, we conduct comprehensive experiments to evaluate the performance of the proposed HASCom framework. We compare HASCom against traditional digital communication schemes and state-of-the-art semantic-only communication systems. The evaluation focuses on three key aspects: semantic intelligibility under noisy conditions, preservation of affective fidelity, and the advantages of the heterogeneous design.

### 6.1. Experimental Setup

#### 6.1.1. Datasets and Preprocessing

We use the Emotional Speech Dataset (ESD) [25]. This high-quality multi-speaker dataset is specifically designed for speech synthesis and emotion conversion tasks. The ESD dataset comprises recordings from 10 native English speakers and 10 native Chinese speakers, covering five distinct emotion categories: neutral, happy, angry, sad, and surprise. It collected 350 recordings from each speaker for every emotion. This rich diversity ensures that the model learns robust generalized representations across varied linguistic contents and emotional prosodies.

For data preprocessing, all audio recordings are resampled to 16 kHz to ensure consistency across the system. We extract 80-dimensional Mel-spectrograms as acoustic features using the Short-Time Fourier Transform (STFT). The STFT configuration includes a 1024-point FFT size, a 256-point hop length, and a 1024-point Hann window. These Mel-spectrograms serve as the ground truth targets for the generative diffusion decoder. We also employ the Montreal Forced Aligner (MFA) to extract phoneme-level alignments from the raw audio, which serve as input to the semantic coding stream.

The proposed HASCom framework is implemented using the PyTorch 2.0.0 library and trained on an NVIDIA RTX 4090 GPU with 24 GB of video memory. The network parameters were optimized using the Adam optimizer with $\beta_{1}=0.9$, $\beta_{2}=0.998$, and an epsilon value of $10^{-9}$. A constant learning rate of $1\times 10^{-4}$ was used throughout the training process to ensure the stable convergence of the joint source-channel coding module. The batch size was set to 32 to accommodate the memory constraints and maintain accurate gradient estimation.

For the conditional diffusion decoder, the total number of diffusion steps $T_{train}$ is set to 2000 during training to capture the fine-grained acoustic details of the Mel-spectrograms. During the inference phase, we use a fast sampling strategy with $T_{infer}=50$ steps to reduce computational latency. We apply a temperature parameter τ=1.3 to the stochastic noise generator to enhance the expressiveness of the synthesized speech. To enhance the robustness of the system against varying channel conditions, the model was trained under a dynamic SNR environment, where the signal-to-noise ratio was uniformly sampled from [0,20] dB. The detailed hyperparameter settings are summarized in Table 3.

#### 6.1.2. Simulation Assumptions

To clearly define the boundaries of our experimental evaluation, we enumerate the key simplifying assumptions made in this study:

1. Channel Models: We assume block-fading characteristics for both the AWGN and Rayleigh fading channels. For the Rayleigh scenario, we assume the receiver has access to perfect Channel State Information (CSI) to perform ideal Zero-Forcing (ZF) equalization.
2. Power Constraints: The average transmit power for the analog JSCC stream is strictly normalized to P=1 per symbol.
3. Bandwidth Allocation: The bandwidth allocation ratio between the digital semantic link and the analog affective link is assumed to be a fixed hyperparameter determined by the extracted feature dimensions, without dynamic bandwidth allocation (DBA) based on instantaneous channel conditions.
4. Quantization Bit-Depth: The discrete semantic features are quantized using a fixed 8-bit depth prior to the LDPC encoding process.
5. Data Constraints: The evaluation is currently constrained to the clean, studio-quality speech environments provided by the ESD dataset. The speakers and languages (English and Chinese) are bounded by this dataset, meaning the system is evaluated under controlled emotional acting without extreme, in-the-wild background noise outside of the simulated wireless channel impairments.

Furthermore, the detailed architectural dimensions and parameter counts of the proposed HASCom framework are summarized in Table 4.

#### 6.1.3. Baselines

To accurately evaluate HASCom’s performance, we compare it against three representative semantic communication baselines. To ensure absolute fairness and resolve previous inconsistencies, we explicitly confirm that all baseline models are evaluated consistently across both objective and subjective metrics, under identical channel conditions, signal-to-noise ratios, and preprocessing protocols as our proposed framework.

**DeepSC-SR (Deep Semantic Communication for Speech Reconstruction):** This baseline represents the state-of-the-art JSCC-based speech-semantic communication system. It employs a monolithic encoder–decoder architecture to map speech features directly to continuous analog channel symbols. It focuses on minimizing the end-to-end reconstruction loss without explicit disentanglement of semantic and affective features.

**SE-DeepSC (Speech Enhancement + DeepSC):** This baseline represents a Denoise-then-Transmit cascaded strategy designed to mitigate environmental noise. A dedicated Speech Enhancement (SE) module is deployed at the transmitter to filter out background noise from the raw waveform before the clean speech is fed into the DeepSC encoder.

**DeepSC-S-SE (DeepSC + Speech Enhancement):** This benchmark represents a strategy of transmitting data first and then performing noise reduction. The speech is first transmitted and reconstructed via the standard DeepSC system, then an SE module is applied at the receiver end as a post-processor to suppress channel-induced noise and artifacts in the synthesized speech.

#### 6.1.4. Channel Models

We simulated the physical layer transmission scenarios read under both Additive White Gaussian Noise (AWGN) and Rayleigh fading environments. During the evaluation phase, the signal-to-noise ratio (SNR) ranged from 0 decibels to 18 decibels, covering conditions from extreme interference to high-quality channels.

### 6.2. Performance Evaluation Metrics

We use a comprehensive set of indicators to assess the reliability of the physical layer, the consistency of semantics, the objective waveform distortion, and the subjective quality of perception:

**Feature Reconstruction MSE:** We use the Mean Squared Error (MSE) to evaluate the reconstruction quality of the transmitted features. This metric calculates the squared difference between the original feature vectors generated by the encoder and the noisy features recovered by the decoder. A lower MSE value means that the system can effectively recover the semantic and emotional features even under noisy channel conditions.

**Similarity Score:** This metric measures the semantic and emotional consistency between the original and the reconstructed speech. It is computed based on the cosine similarity between the source emotion embeddings and the received ones. A higher similarity score indicates that the meaning and emotional intent of the speaker are well preserved during the communication process.

**Mel-Cepstral Distortion (MCD):** MCD is a quantitative objective metric that measures the spectral distance between the synthesized speech and the ground-truth recordings. It utilizes Dynamic Time Warping (DTW) to align the acoustic sequences frame by frame. A lower MCD value generally indicates higher spectral fidelity and a more accurate reconstruction of complex harmonic structures.

**Perceptual Evaluation of Speech Quality (PESQ):** PESQ is a standard intrusive objective metric originally designed to evaluate the waveform quality of traditional communication systems. It assesses the degradation of the reconstructed speech waveform compared to the original clean signal. While a higher PESQ score traditionally indicates better waveform preservation, it strictly demands precise microscopic phase alignment and exact temporal synchronization.

**Short-Time Objective Intelligibility (STOI):** STOI is a highly correlated objective metric used to evaluate the intelligibility of the reconstructed speech. It computes the correlation of short-time temporal envelopes between the clean reference and the degraded signal. The STOI score ranges from 0 to 1, with a higher value indicating superior speech clarity and structural intelligibility.

**Mean Opinion Score (MOS):** MOS is a subjective metric used to evaluate how natural the speech sounds to human ears. In our experiments, human listeners were invited to rate the generated audio samples on a scale from 1 (Bad) to 5 (Excellent). A higher MOS score indicates that the reconstructed speech sounds clearer and closer to natural.

### 6.3. Results and Analysis

#### 6.3.1. Training Convergence Analysis of Analog Transmission

**Figure 2:**
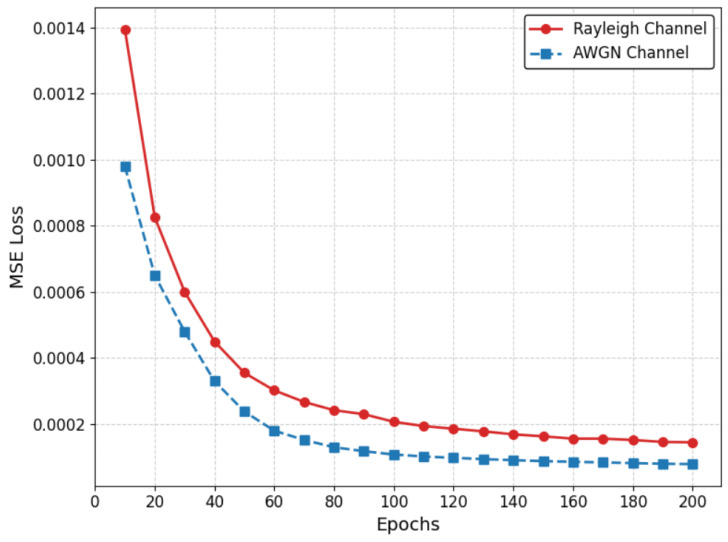
The training convergence curves of the Deep Analog JSCC module under AWGN and Rayleigh fading channels.

To validate the learnability and optimization stability of the proposed Deep Analog Transmission module under different wireless environments, we monitor the training loss curves of the JSCC encoder–decoder network under both AWGN and Rayleigh fading channels. The convergence behaviors are shown in Figure 2, which depicts the Mean Squared Error (MSE) loss of the affective feature reconstruction over 200 training epochs.

These curves illustrate the optimization efficiency of the heterogeneous channels. As shown in Figure 2, both channels exhibit a sharp drop in training loss during the first 20 epochs. For the ideal AWGN channel, the loss decreases from approximately $1.0\times 10^{-3}$ to $3.3\times 10^{-4}$. In the more challenging Rayleigh fading scenario, the initial loss is naturally higher at around $1.4\times 10^{-3}$, but it still rapidly descends to $4.5\times 10^{-4}$. This indicates that the feature adapter and the JSCC encoder can efficiently learn to map high-dimensional emotions and speakers into a compact and robust simulated constellation, without encountering the problem of gradient vanishing.

From the 30th cycle to the 200th cycle, the errors for both channels show a monotonically and steadily decreasing trend. The AWGN curve eventually converges to a very small noise baseline of approximately $7.9\times 10^{-5}$. For the Rayleigh channel, the model converges to approximately $1.45\times 10^{-4}$. Although the final convergence loss under Rayleigh fading is slightly higher due to the inherent deep fading dips of the multipath environment, no obvious oscillations or peaks were observed in either curve. This confirms the stability of the proposed architecture. This result theoretically guarantees that the simulated channel can provide accurate emotional features resilient to varying and dynamic channel impairments.

#### 6.3.2. Semantic Consistency Analysis

We use the similarity metric to evaluate the semantic consistency retention ability of this system. Before analyzing the results, we formally define this Similarity Score to ensure experimental reproducibility. Rather than relying on external post-processing evaluation networks, this metric directly quantifies the structural fidelity of the deep neural representations transmitted over the noisy physical channel. Let $Z_{a}$ denote the flattened, one-dimensional original continuous latent vector (comprising the packed speaker and emotion embeddings) prior to the JSCC encoder, and let ${\hat{Z}}_{a}$ denote the corresponding reconstructed latent vector at the output of the JSCC decoder. The Similarity Score is mathematically formulated as the percentage-normalized cosine similarity between these two high-dimensional vectors:

(28) $$\mathrm{Similarity}=\frac{Z_{a}\cdot {\hat{Z}}_{a}}{∥Z_{a}{∥}_{2}{∥{\hat{Z}}_{a}∥}_{2}}\times 100\%.$$

**Figure 3:**
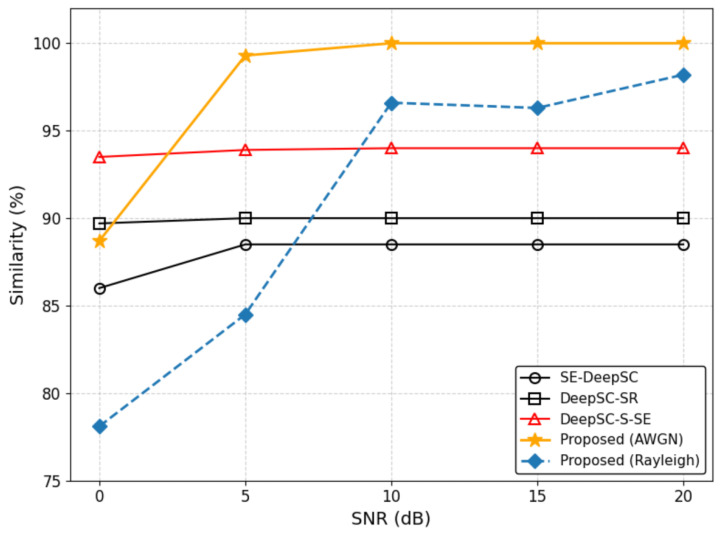
Performance comparison of multiple models in terms of Similarity under AWGN and Rayleigh fading channels.

This metric objectively isolates and evaluates the system’s robustness in recovering the continuous paralinguistic features against channel impairments. Figure 3 compares the performance of the proposed HASCom framework against three semantic communication baselines: DeepSC-SR, SE-DeepSC, and DeepSC-S-SE.

As shown in Figure 3, the baseline methods suffer from a performance ceiling, saturating between 88% and 94%. This limitation arises because they rely on end-to-end analog transmission for the entire speech representation, where channel noise inevitably degrades the discrete linguistic information.

However, the HASCom framework exhibits a distinctly superior performance trajectory. Under the AWGN channel, while the similarity score dips to approximately 89% in the low SNR regime (0 dB)—slightly lower than the DeepSC-S-SE baseline due to residual bit errors before the digital channel coding fully converges—a sharp performance leap is observed as the SNR improves. Once the SNR exceeds 5 dB, surpassing the digital channel’s decoding threshold, the similarity score rapidly rises to nearly 100%, significantly outperforming all baselines.

Furthermore, to validate the system’s robustness in complex practical scenarios, we evaluated HASCom under the challenging Rayleigh fading channel. As depicted by the dashed line, the severe multipath fading inevitably causes a performance drop in the extremely low SNR regime, dipping to approximately 78% at 0 dB. Nevertheless, as the channel conditions improve (SNR ≥ 10 dB), the similarity swiftly recovers to over 96% and progressively approaches the AWGN upper bound.

The sustained superiority of HASCom is directly attributed to its heterogeneous transmission strategy, where the rigid linguistic text is protected by a reliable digital link, while the fluid emotion and timbre are adaptively conveyed through the JSCC stream. This confirms our model can achieve near-error-free semantic transmission under moderate to good channel conditions and maintain excellent anti-interference capabilities even in harsh fading environments.

#### 6.3.3. Perceptual Quality Evaluation

**Figure 4:**
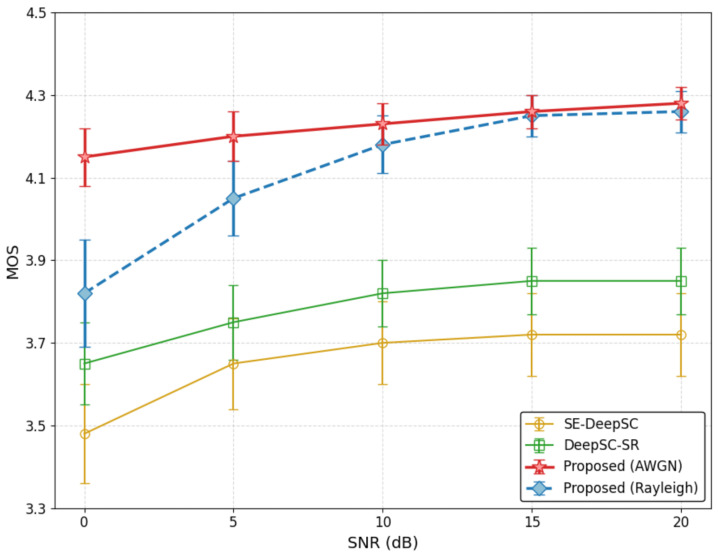
Performance comparison of multiple models in terms of MOS under AWGN and Rayleigh fading channels. Error bars indicate 95% confidence intervals.

We finally evaluate the subjective perceptual quality of the synthesized speech using the Mean Opinion Score (MOS). To ensure the rigorousness, fairness, and reproducibility of the subjective evaluation, we established a standardized double-blind listening protocol. A group of 25 proficient listeners (including native English and Chinese speakers to match the ESD dataset) was recruited. We randomly selected 50 distinct text utterances covering all five emotion categories. For each condition, the synthesized audio samples were presented to the listeners in a completely randomized order to prevent any sequence bias. The listeners were completely blind to the underlying generation models and channel conditions. They were instructed to rate the audio samples on a standard 5-point Absolute Category Rating (ACR) scale (1 = Bad, 2 = Poor, 3 = Fair, 4 = Good, 5 = Excellent) based on three criteria: linguistic intelligibility, naturalness of the voice, and emotional expressiveness. To validate the reliability of the subjective scores, we computed Krippendorff’s alpha (α), achieving an inter-rater agreement score of α=0.82, which indicates high consensus among the evaluators. All reported MOS values include 95% Confidence Intervals (CIs) to represent statistical significance. Figure 4 presents the MOS results from human listening tests, comparing the proposed HASCom against the SE-DeepSC and DeepSC-SR baselines.

Consistent with the objective semantic consistency results, the proposed framework significantly outperforms the baselines in terms of speech naturalness and intelligibility.

As observed, the pure analog baselines (SE-DeepSC and DeepSC-SR) fluctuate between a MOS of 3.48 and 3.85. These methods often suffer from residual channel noise, resulting in audible artifacts and robotic prosody, particularly in low SNR conditions where their confidence intervals (error bars) are noticeably wider.

In sharp contrast, under the AWGN channel, HASCom achieves a remarkable MOS of 4.15 even at 0 dB. This superior performance is primarily driven by our heterogeneous transmission strategy: the digital linguistic stream guarantees accurate content reconstruction, ensuring high intelligibility, while the robust JSCC stream effectively preserves the paralinguistic features. Furthermore, as the SNR improves, the MOS of our method stabilizes around 4.28, approaching ground-truth quality with narrow confidence intervals.

To further demonstrate the system’s robustness against complex wireless environments, we additionally evaluated the subjective quality under the Rayleigh fading channel. As depicted by the dashed line, severe multipath fading inevitably degrades the perceptual quality at 0 dB, dropping the MOS to approximately 3.82 with an increased variance. However, it is noteworthy that our system under Rayleigh fading still outperforms the baseline methods operating under the ideal AWGN channel. More importantly, as the SNR increases beyond 10 dB, the MOS rapidly recovers and closely tracks the AWGN upper bound, reaching over 4.25.

This indicates that the conditional diffusion decoder successfully utilizes the robust semantic cues to synthesize high-fidelity waveforms, effectively decoupling the perceptual quality from dynamic channel fluctuations.

#### 6.3.4. Analysis of Mel-Spectrogram Characteristics

**Figure 5:**
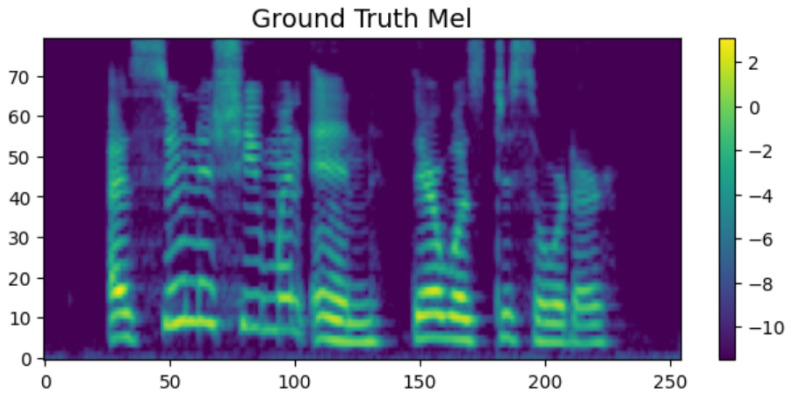
Ground Truth Mel-spectrogram: The original target spectrogram derived from the source speech.

**Figure 6:**
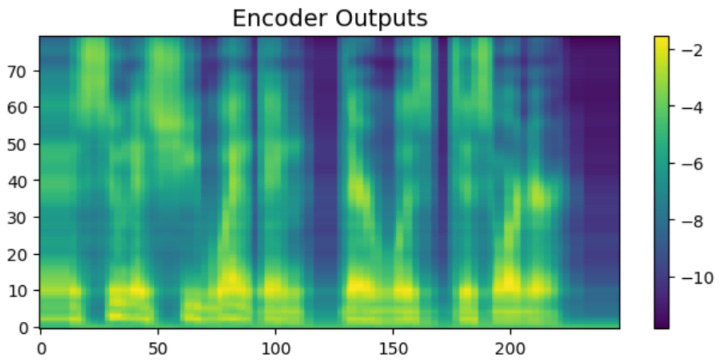
The latent representation generated by the text encoder before diffusion.

**Figure 7:**
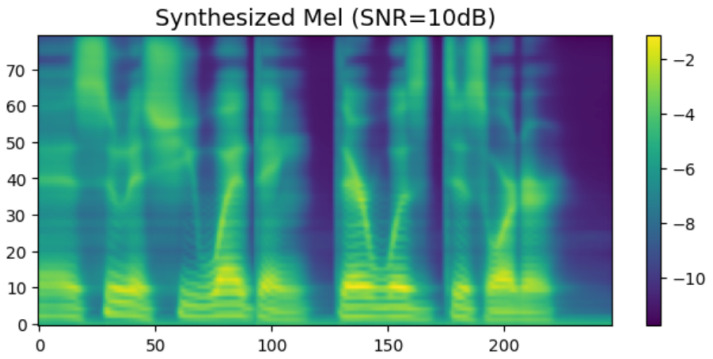
The reconstructed spectrogram generated by the diffusion model under the AWGN channel.

**Figure 8:**
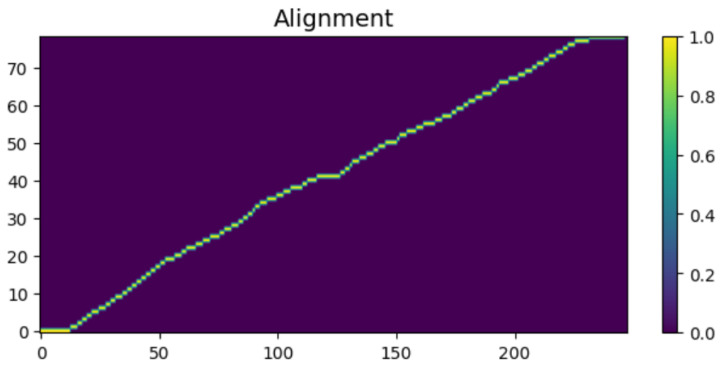
The alignment path between text characters and acoustic frames under the AWGN channel.

**Figure 9:**
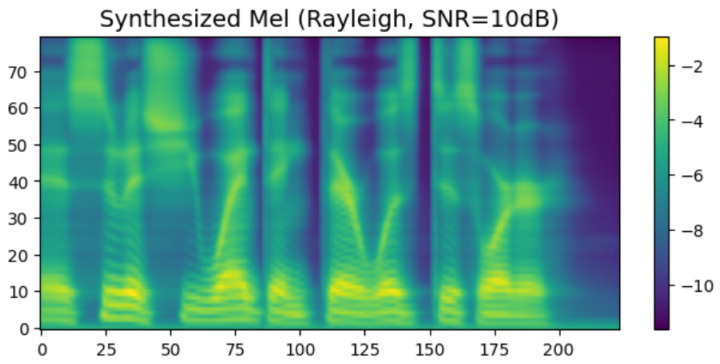
The reconstructed spectrogram generated by the diffusion model under the Rayleigh fading channel (SNR = 10 dB).

**Figure 10:**
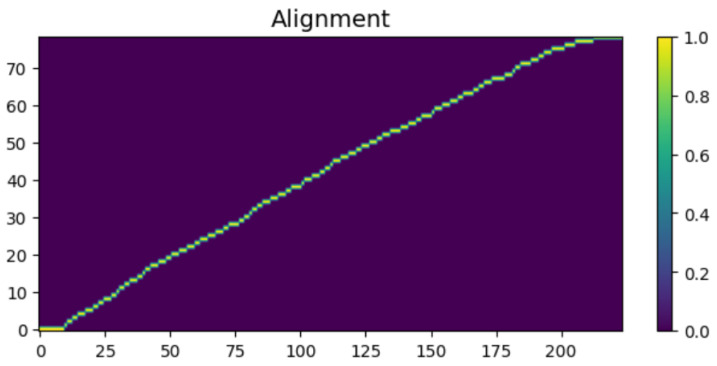
The robust alignment path maintained under the Rayleigh fading channel (SNR = 10 dB).

To intuitively verify the generation capability of the proposed HASCom framework under varying channel conditions, we visualize the intermediate representations and the final synthesized output. Figure 5, Figure 6, Figure 7, Figure 8, Figure 9 and Figure 10 present the progression of the speech generation process, comparing the Ground Truth, the Encoder Output, the Synthesized Mel-spectrograms, and the Alignment maps.

As illustrated, the generated mel-spectrograms demonstrate the progression of the diffusion model output at different stages. First, regarding the Linguistic Prior, the output of the text encoder in Figure 6 exhibits a smoothed and averaged texture. This is within our expectations, as the encoder predicts the overall language distribution based on the text input, providing a basic representation without fine-grained rhythmic variations.

The system demonstrates Robust Alignment as shown in Figure 8. The clear, unbroken diagonal line in the alignment map indicates that the system maintains robust synchronization between text characters and acoustic frames. More importantly, as evidenced by Figure 10, this strictly monotonic alignment is perfectly maintained even when subjected to the severe multipath interference of the Rayleigh fading channel at 10 dB. This proves that our digital semantic link and structural prior network provide an unbreakable backbone for speech synchronization, ensuring the coherence and stability of speech regardless of harsh channel impairments.

The final Expressive Synthesis is visualized in Figure 7 and Figure 9. By integrating the semantic and emotional features of the transmission, the conditional diffusion decoder significantly enriches the spectral representation. Compared with the output of the encoder, the synthesized spectral graph in Figure 7 restores rich high-frequency details, clear harmonic structures, and subtle pitch variations. Furthermore, under the challenging Rayleigh fading conditions (Figure 9), the model still successfully reconstructs these highly complex acoustic textures. Although minor spectral smoothing can be observed in certain high-frequency bands compared to the ideal AWGN conditions due to deep fading dips, the overall harmonic integrity and formant transitions remain highly consistent with the ground truth. This result visually confirms that our proposed model can generate highly natural and emotionally expressive speech with strong resilience to dynamic channel noise.

#### 6.3.5. Quantitative Objective Metrics Analysis

To supplement our evaluation, we assessed traditional objective metrics: Mel-Cepstral Distortion (MCD), Perceptual Evaluation of Speech Quality (PESQ), and Short-Time Objective Intelligibility (STOI).

**Figure 11:**
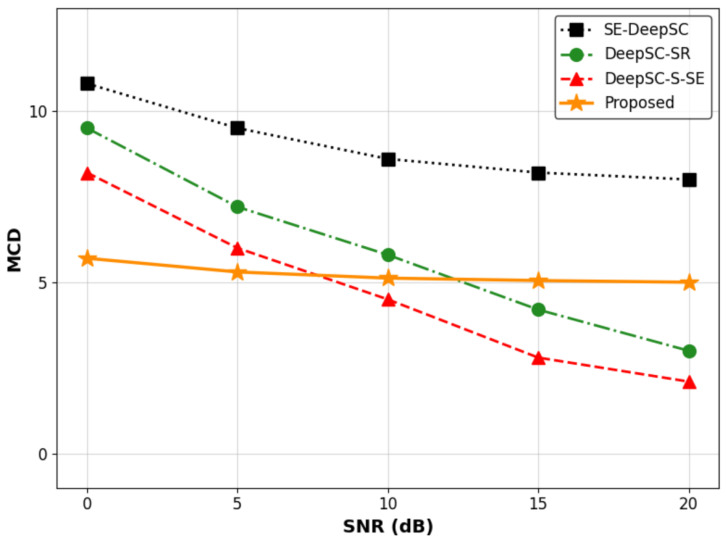
MCD performance under various SNR conditions.

As depicted in Figure 11, the proposed framework exhibits remarkable MCD stability. In severe noise conditions (SNR = 0 dB), our model achieves an MCD of ≈5.7, largely avoiding the severe degradation seen in the traditional baselines. However, at high SNRs, while MSE-based models reach lower MCDs due to their strict waveform replication, our proposed model maintains a stable MCD of ≈5.0. Concurrently, the calculated PESQ value remains at approximately 1.7. Similarly, the STOI score records approximately 0.74 at 0 dB and exhibits only a marginal improvement as the SNR increases, stabilizing at roughly 0.75.

We attribute these numerical plateaus to the inherent nature of our diffusion-based generative receiver. Rather than performing sample-by-sample mathematical filtering, our system synthesizes entirely new high-fidelity waveforms from deep latent representations. The autonomous duration prediction naturally introduces microscopic phase differences, temporal shifts, and prosodic deviations from the ground-truth recordings. Because classical metrics like MCD, PESQ, and STOI are intrusive algorithms relying on strict frame-by-frame DTW, exact temporal synchronization, and phase alignment, they inflict severe numerical penalties on these perceptually natural variations. While generative models prioritize perceptual naturalness over making a rigid waveform-preserving copy, we honestly acknowledge this objective performance gap as a shortcoming. Future work will explore advanced duration modeling and phase-aware generation to bridge this objective–subjective gap without sacrificing generative diversity.

#### 6.3.6. Ablation Study

To rigorously validate the necessity of the proposed heterogeneous sub-modules, we conducted a quantitative ablation study at an SNR of 10 dB. Table 5 presents the Mel-Cepstral Distortion (MCD), Perceptual Evaluation of Speech Quality (PESQ), and Short-Time Objective Intelligibility (STOI) scores under various system configurations. A lower MCD value indicates a smaller spectral distance, while higher PESQ and STOI values reflect better perceptual waveform quality and intelligibility, acknowledging the inherent numerical penalty imposed on generative models due to phase mismatch.

As shown in Table 5, the proposed full model achieves the best performance with the lowest MCD of 5.12 dB, the highest PESQ of 1.78, and a peak STOI of 0.742. This indicates the highest spectral fidelity, the most accurate reconstruction of complex harmonic structures, and superior speech clarity.

Stripping the emotion embedding increases the MCD to 5.45 dB and drops the PESQ to 1.65 and the STOI to 0.715. Without emotional guidance, the model fails to capture dynamic pitch transitions (F0 contours) and high-frequency energy variations, causing the speech to lose its expressive prosody. Conversely, removing the speaker embedding further degrades the performance (MCD = 5.58 dB, PESQ = 1.58, STOI = 0.698) because the network struggles to reconstruct the specific formant distributions of the target timbre.

Finally, disabling the entire JSCC analog stream (Pure Semantic Base) yields the highest distortion, with the MCD deteriorating to 5.85 dB, the PESQ falling to 1.50, and the STOI dropping to 0.675. This significant numerical penalty reflects severe spectral over-smoothing and the loss of fine acoustic textures, which directly corresponds to a robotic, unnatural voice with reduced intelligibility.

This quantitative evidence firmly justifies our dual-stream architecture: the digital semantic link provides structural boundaries, while the analog affective stream is indispensable for rendering expressive, personalized acoustic details and maximizing generation quality. Furthermore, to observe the system’s behavior across different channel qualities, we briefly analyzed the ablation trends at extreme SNR boundaries (0 dB and 20 dB). At a low SNR (0 dB), the analog JSCC stream experiences fading, which reduces affective rendering quality. However, the LDPC-protected digital semantic stream still ensures the recovery of basic linguistic content. Conversely, at a high SNR (20 dB), the channel introduces minimal distortion. In this condition, the inclusion of the emotion and speaker embeddings effectively reduces spectral distortion and improves the overall synthesis quality. These trends indicate that the dual-stream architecture maintains basic intelligibility at low SNRs while achieving better acoustic performance at high SNRs.

#### 6.3.7. Computational Complexity and Inference Latency

To comprehensively evaluate the deployment feasibility of the proposed HASCom framework, we quantify its overall computational complexity (number of parameters) and measure the inference latency across different hardware platforms. The processing efficiency is evaluated using the Real-Time Factor (RTF), defined as the ratio of the total processing time to the duration of the synthesized speech audio.

Table 6 summarizes the trainable parameters, module-wise inference latency, and the RTF evaluated on two commercial GPUs, namely an NVIDIA RTX 4090 (24GB) and an estimated equivalent for the NVIDIA RTX 3080 Ti (12GB), generating a 0.546-s speech segment.

As detailed in Table 6, the total parameter count of the HASCom system is approximately 63.23 million. A critical observation from the latency breakdown is the ultra-high efficiency of the proposed deep analog transmission modules. Both the JSCC Encoder and Decoder require merely 0.08 million parameters and exhibit negligible inference latencies (approximately 0.08 ms on the RTX 4090), which firmly proves that our heterogeneous transmission scheme introduces virtually zero delay to the communication pipeline.

The bulk of the computational overhead is concentrated at the receiver side, specifically within the Grad-TTS conditional diffusion decoder. Due to the iterative reverse denoising process configured at 50 steps, it consumes 941.63 ms on the RTX 4090, resulting in a total system RTF of 1.732 (and an estimated RTF of 3.030 on the RTX 3080 Ti). While this currently operates in a near-real-time regime, this latency trade-off is typical for high-fidelity generative models. In practical deployments, this generation delay can be substantially mitigated by integrating advanced fast-sampling algorithms (e.g., DPM-Solver or Consistency Models) to reduce the required diffusion steps from 50 to under 5, which would easily push the entire semantic communication system well below the real-time threshold (RTF <1.0).

## 7. Conclusions

In this paper, we propose the Heterogeneous Affective Speech Semantic Communication System (HASCom), which is a novel framework capable of effectively balancing the trade-off between semantic accuracy and emotional fidelity. Unlike homogeneous transmission schemes, HASCom achieves this balance by decoupling the speech signal into discrete semantic streams and continuous emotional streams, intelligently selecting the appropriate channel based on the physical properties of the features. We have constructed a hybrid digital–analog transmission architecture to ensure strict language alignment while retaining the infinite-resolution emotional cues. Additionally, at the receiving end, we have designed a prior-guided conditional diffusion model that reconstructs high-quality speech by adding emotional details on top of the semantic prior. Experimental results demonstrate that the proposed method achieves superior performance in both objective and subjective evaluations compared to existing baselines. In terms of semantic preservation, the system achieves a semantic similarity score approaching 100% under stable channel conditions, significantly outperforming DeepSC-S. Regarding perceptual quality, HASCom achieves a Mean Opinion Score (MOS) of approximately 4.3, demonstrating its ability to generate natural, emotionally expressive speech even in noisy environments.

Despite these promising results, we acknowledge several limitations in the current study. First, the experimental evaluation is primarily restricted to a single dataset (ESD), which may not fully capture the acoustic diversity of in-the-wild, multi-lingual, or conversational speech. Second, while our evaluation incorporates both AWGN and Rayleigh fading models, the system has yet to be tested on physical Software-Defined Radio (SDR) testbeds under real-world, dynamic radio frequency interference. Finally, although we have quantified the hardware inference latency, the current 50-step diffusion generation process still poses computational challenges for strict real-time edge deployment, and more comprehensive ablation studies on extreme channel conditions are required to fully isolate the theoretical performance bounds of each sub-module.

In future work, we aim to extend our heterogeneous framework to support real-time multi-speaker scenarios, validate the system across more diverse datasets and physical testbeds, and investigate lightweight fast-sampling adaptation strategies (e.g., DPM-Solver) for deployment on resource-constrained edge devices. 

## Figures and Tables

**Table 1:** Structured Comparison of the Proposed HASCom Framework with Closest Prior Works.

Study	Model Type	Transmission Strategy	Affective Handling	Channel Model	Reconstruction Backbone	Key Limitations
DeepSC-S [17]	Speech SemCom	Homogeneous Analog (Deep JSCC)	Ignored	AWGN Rayleigh	MSE-based Neural Decoder	Loss of paralinguistic tone; muffled reconstruction quality.
Diffusion models for audio semantic communication [20]	Audio SemCom	Homogeneous Analog (Deep JSCC)	Ignored	AWGN	Unconditional Diffusion Model	Highly vulnerable to channel noise; suffers from semantic hallucinations.
EESC-S [19]	Emotion-Aware SemCom	Homogeneous Analog (Concatenated features)	Integrated (Coupled)	AWGN Rayleigh	MSE-based Neural Decoder	Suboptimal bandwidth use; semantic content is vulnerable to cliff-effects.
Proposed HASCom	Affective Speech SemCom	Heterogeneous (Digital LDPC Analog JSCC)	Decoupled Preserved	AWGN Rayleigh	Prior-Guided Conditional Diffusion	High inference latency; evaluated on a single dataset; lacks real-world edge deployment testing.

**Table 2:** Summary of Key Mathematical Notations.

Symbol	Definition	Symbol		Definition
Signals and Latent Representations		Wireless Channel Model		
$W,\hat{W}$	Raw input speech waveform and the reconstructed waveform		$S_{c},Y_{c}$	Transmitted and received digital symbols
$X_{0},X_{t},X_{T}$	Ground-truth Mel-spectrogram, noisy state at time *t*, and terminal state		$S_{a},Y_{a}$	Transmitted and received analog JSCC symbols
$Z_{c},{\hat{Z}}_{c}$	Extracted and recovered discrete semantic (linguistic) representations		$h_{c},h_{a}$	Fading coefficients for digital and analog channels
$Z_{e},Z_{s}$	Extracted emotion and speaker embeddings		$n_{c},n_{a}$	Additive White Gaussian Noise (AWGN) vectors
$Z_{a},{\hat{Z}}_{a}$	Unified continuous affective latent vector and its recovered version		*P*	Average transmission power constraint
Neural Network Functions		Diffusion and Alignment Dynamics		
$E_{c}$	Semantic content encoder (Wav2Vec 2.0 backbone)		$\mu ,\tilde{\mu }$	Aligned and unaligned semantic structural prior (mean)
$E_{e},E_{s}$	Emotion and speaker encoders (WavLM backbone)		$\beta_{t}$	Non-negative noise schedule at diffusion time *t*
$E_{jscc},D_{jscc}$	Deep JSCC analog encoder and non-linear decoder		$A^{*}$	Optimal monotonic alignment path derived via MAS
$P_{prior}$	Structural Prior Network		$d_{mas},d_{pred}$	Target phoneme durations and network-predicted durations
$\epsilon_{\theta }$	Neural score estimator (Affective-conditioned diffusion U-Net)			
Optimization Objectives				
$L_{total}$	End-to-end composite loss function		$L_{s},L_{t}$	Structural alignment loss and temporal duration loss
$L_{d},L_{a}$	Diffusion denoising loss and analog affective consistency loss		$\lambda_{a},\lambda_{s}$	Hyperparameters balancing affective consistency and semantic alignment

**Table 3:** Hyperparameter settings of the proposed framework.

Parameter	Value	Parameter	Value
Total Epochs	200	Optimizer	Adam
Learning Rate	$1\times 10^{-4}$	Batch Size	32
Training Steps ($T_{train}$)	2000	Inference Steps ($T_{infer}$)	50
Min. Noise Level ($\beta_{min}$)	0.05	Max. Noise Level ($\beta_{max}$)	20.0
Temperature (τ)	1.3	Length Scale	0.91
$\lambda_{a}$	1.0	$\lambda_{s}$	1.0
Channel Type	AWGN & Rayleigh	Training SNR	U(0,20) dB
FFT Size	1024	Hop Length	256

**Table 4:** Detailed Architectural Dimensions and Parameter Counts of the HASCom Framework.

Module	Layer/Operation	Output Dimension	Parameters (Approx.)
Analog Affective Link (Deep JSCC)			
Feature Adapter	Input (Speaker + Emotion embeddings)	[B, 1, 192] + [B, 1, 768]	0
	Reshape & Concatenation	[B, 5, 192]	0
JSCC Encoder	Input Sequence	[B, 5, 192]	–
	Linear → LayerNorm → ReLU	[B, 5, 256]	49,920
	Linear (Channel Projection)	[B, 5, 128]	32,896
	Total Encoder		≈0.08 M
JSCC Decoder	Input from Wireless Channel	[B, 5, 128]	–
	Linear → LayerNorm → ReLU	[B, 5, 256]	33,536
	Linear (Feature Restoration)	[B, 5, 192]	49,344
	Total Decoder		≈0.08 M
Conditioned Diffusion U-Net (Neural Score Estimator)			
Time Embedding	Sinusoidal Positional Embedding	[B, 80]	0
	MLP (Linear → Mish → Linear)	[B, 80]	32,400
Down-Blocks (×3)	2D ResNet Block + Time Injection	[B, $C_{out}$, $H/2^{i}$, $W/2^{i}$]	≈6.5 M
	Emotion Cross-Attention (i<2)	[B, $C_{out}$, $H/2^{i}$, $W/2^{i}$]	≈14.8 M
	Affective FiLM Modulation	[B, $C_{out}$, $H/2^{i}$, $W/2^{i}$]	≈0.5 M
	Linear Attention & Downsample 2D	[B, $C_{out}$, $H/2^{i+1}$, $W/2^{i+1}$]	≈1.2 M
Bottleneck (Mid)	2D ResNet Block + Time Injection	[B, 320, 10, W/8]	≈3.8 M
	Linear Attention + Affective FiLM	[B, 320, 10, W/8]	≈0.6 M
	2D ResNet Block + Time Injection	[B, 320, 10, W/8]	≈3.8 M
Up-Blocks (×3)	Concat Skip Connection	[B, $C_{in}\times 2$, $H/2^{i}$, $W/2^{i}$]	0
	2D ResNet Block + Time Injection	[B, $C_{out}$, $H/2^{i-1}$, $W/2^{i-1}$]	≈8.5 M
	Affective FiLM Modulation	[B, $C_{out}$, $H/2^{i-1}$, $W/2^{i-1}$]	≈0.5 M
	Linear Attention & Upsample 2D	[B, $C_{out}$, $H/2^{i-1}$, $W/2^{i-1}$]	≈1.2 M
Final Output	Block → Conv2D (Output Masking)	[B, 80, W]	57,680
	Total Diffusion Parameters		≈32.50 M

**Table 5:** Ablation Study on Heterogeneous Sub-modules (SNR = 10 dB).

System Configuration	MCD (dB)	PESQ	STOI
Proposed Full Model	5.12	1.78	0.742
w/o Emotion (Semantic + Speaker only)	5.45	1.65	0.715
w/o Speaker (Semantic + Emotion only)	5.58	1.58	0.698
Pure Semantic Base (w/o Affective Stream)	5.85	1.50	0.675

**Table 6:** Computational complexity and inference latency across different hardware platforms.

Module	Params (M)	RTX 4090		RTX 3080 Ti	
		Latency (ms)	RTF	Latency (ms)	RTF
JSCC Encoder	0.08	0.08	<0.001	0.14	<0.001
JSCC Decoder	0.08	0.08	<0.001	0.14	<0.001
Grad-TTS (50 steps)	49.14	941.63	1.726	1647.85	3.018
HiFi-GAN Vocoder	13.93	3.50	0.006	6.13	0.011
Total System	63.23	945.29	1.732	1654.26	3.030

## Data Availability

The original contributions presented in this study are included in the article. Further inquiries can be directed to the corresponding author.

## References

[1] Zhao C., Du H., Niyato D., Kang J., Xiong Z., Kim D.I., Shen X., Letaief K.B. (2025). Generative AI for secure physical layer communications: A survey. IEEE Trans. Cogn. Commun. Netw..

[2] Miao J., Wang Z., Wang M., Garg S., Hossain M.S., Rodrigues J.J. (2024). Secure and efficient communication approaches for Industry 5.0 in edge computing. Comput. Netw..

[3] Dong P., Ge J., Wang X., Guo S. (2021). Collaborative edge computing for social internet of things: Applications, solutions, and challenges. IEEE Trans. Comput. Soc. Syst..

[4] He X., Jiang Y., Liu Y., Cui H., Pan H., Mao Y. (2025). Transforming 6G mobile edge intelligence with large models. IEEE Netw..

[5] Xia L., Sun Y., Liang C., Zhang L., Imran M.A., Niyato D. (2025). Generative AI for semantic communication: Architecture, challenges, and outlook. IEEE Wirel. Commun..

[6] Liu Y., Du H., Niyato D., Kang J., Xiong Z., Mao S., Zhang P., Shen X. (2024). Cross-modal generative semantic communications for mobile AIGC: Joint semantic encoding and prompt engineering. IEEE Trans. Mob. Comput..

[7] Qin Z., Tao X., Lu J., Tong W., Li G.Y. (2021). Semantic communications: Principles and challenges. IEEE Netw..

[8] Zhang Q., Saad W., Bennis M., Debbah M. (2024). Generative AI for Physical Layer Communications: A Survey. IEEE Trans. Cogn. Commun. Netw..

[9] Xie H., Ye Z., Li G.Y., Juang B.H.F. (2021). Deep learning enabled semantic communication systems. IEEE Trans. Signal Process..

[10] Al-Hawawreh M., Hossain M.S. (2025). A human-centered quantum machine learning framework for attack detection in IoT-based healthcare Industry 5.0. IEEE Internet Things J..

[11] Yeo Y., Kim J., Song H.-Y. (2024). Enhanced semantic communication schemes for speech signals. Electron. Lett..

[12] Weng Z., Qin Z. (2021). Semantic communication systems for speech transmission. IEEE J. Sel. Areas Commun..

[13] Belin P., Zatorre R.J., Lafaye P., Lioret P., Pike B., Penhune V. (2000). Voice-selective areas in human auditory cortex. Nature.

[14] Han T., Yang Q., Shi Z., He S., Zhang Z. (2022). Semantic-preserved communication system for highly efficient speech transmission. IEEE J. Sel. Areas Commun..

[15] Kumar Y., Koul A., Singh C. (2023). A deep learning approaches in text-to-speech system: A systematic review and recent research perspective. Multimed. Tools Appl..

[16] Obert A., Gunter T.C., Kotz S.A. (2026). Neural integration of affective prosodic and semantic cues in non-literal forms of speech understanding. bioRxiv.

[17] Weng Z., Qin Z., Tao X., Pan C., Liu G., Li G.Y. (2023). Deep learning enabled semantic communications with speech recognition and synthesis. IEEE Trans. Wirel. Commun..

[18] Xiao Z., Yao S., Dai J., Wang S., Niu K., Zhang P. Wireless deep speech semantic transmission. Proceedings of the IEEE International Conference on Acoustics, Speech and Signal Processing (ICASSP).

[19] Tan K., Zhao H., Zhang Y., Cao K., Luo P., Zhang Y., Wei J. (2025). EESC-S: An emotion-enhanced semantic communication framework for speech transmission. IEEE Trans. Cogn. Commun. Netw..

[20] Grassucci E., Marinoni C., Rodriguez A., Comminiello D. Diffusion models for audio semantic communication. Proceedings of the IEEE International Conference on Acoustics, Speech and Signal Processing (ICASSP).

[21] Ho J., Jain A., Abbeel P. Denoising diffusion probabilistic models. Proceedings of the 34th International Conference on Neural Information Processing Systems (NIPS 2020).

[22] Baevski A., Zhou H., Mohamed A., Auli M. wav2vec 2.0: A framework for self-supervised learning of speech representations. Proceedings of the NIPS’20: Proceedings of the 34th International Conference on Neural Information Processing Systems.

[23] Chen S., Wang C., Chen Z., Wu Y., Liu S., Chen Z., Li J., Kanda N., Yoshioka T., Xiao X. (2022). WavLM: Large-scale self-supervised pre-training for full stack speech processing. IEEE J. Sel. Top. Signal Process..

[24] Ding Y., Xu Y., Li J., Yang Q., Yuan Z. (2025). Design and performance evaluation of BILCM-ID system with improved stopping criterion. IEEE Trans. Veh. Technol..

[25] Zhou K., Sisman B., Liu R., Li H. (2022). Emotional voice conversion: Theory, databases and ESD. Speech Commun..

